# The Sweating Sickness in England
*Hecker's Epidemics of the Middle Ages. Translated by B. G. Babington, M.D. Sydenham Society Edition. London: 1844.John Caius, M.D. A Boke or Counseill against the Sweat. London: 1552.John Caius. De Ephemerâ Britannicâ. Reprint. London: 1721.State Papers published by Royal Commission. 1830.Hall. Vnion of the two Noble and Illustre Famelies of Lancastre and Yorke. 1548.Grafton's Chronicle. 1569.Stow's Chronicle, by Howes. London: 1611.Fabian's Chronicle. London: 1559.Hollinshed's Chronicle. London: 1587.Chronicle of the Grey Friars of London. Edited by J. G. Nichols. Camden Society. 1852.Diary of Henry Machyn, Citizen of London. Edited by J. G. Nichols. Camden Society. 1848.Owen and Blakeway's History of Shrewsbury. London: 1825.Collection of English Topographical Histories. Various.Sir H. Ellis. Original Letters. London: 1824.Letters of the Kings of England. By J. O. Halliwell, F.S.A. London: 1848.Harleian Manuscripts.Cottonian Manuscripts. Titus, b. xi.Lord Bacon's History of Henry VII. Op. b. iii. London: 1740.Anthony à Wood. History and Antiquities of University, Oxon. 1674.Publications of the Parker Society. 1846-53.


**Published:** 1857-07

**Authors:** Francis C. Webb


### SANITARY REVIEW.

JULY 1857.

### THE SWEATING SICKNESS IN ENGLAND.*

THERE are few subjects which exhibit more points of interest to the epidemiologist and medical historian, than that series of epidemics, of the fifteenth and sixteenth centuries, which went by the name of English Sweating Sicknesses. We are chiefly indebted to a learned German professor, Dr. Hecker, and to his translator Dr. Babington, for the acquaintance we in the present day have with these events; and we would here observe that, in whatever light we may view Professor Hecker's deductions and theories, there can be but one opinion as to his faithfulness and diligence as a medical historian. As his work, however, is published by a society, and is therefore of somewhat limited circulation, we have thought a short historical sketch, embodying, and in some instances slightly amplifying,

* HECKER's Epidemics of the Middle Ages. Translated by B. G. BABINGTON, M.D. Sydenham Society Edition. London: 1844.
JOHN CAIUS, M.D. A Boke or Counseill against the Sweat. London: 1552.
JOHN CAIUS. De Ephemerâ Britannicâ. Reprint. London: 1721.
State Papers published by Royal Commission. 1830.
HALL. Vnion of the two Noble and Illustre Famelies of Lancastre and Yorke. 1548.
GRAFTON's Chronicle. 1569.
STOW's Chronicle, by HOWES. London: 1611.
FABIAN's Chronicle. London: 1559.
HOLLINSHED's Chronicle. London: 1587.
Chronicle of the Grey Friars of London. Edited by J. G. NICHOLS. Camden Society. 185[illegible].
Diary of Henry Machyn, Citizen of London. Edited by J. G. NICHOLS. Camden Society. 1848.
OWEN and BLAKEWAY's History of Shrewsbury. London: 1825.
Collection of English Topographical Histories. Various.
Sir H. ELLIS. Original Letters. London: 1824.
Letters of the Kings of England. By J. O. HALLIWELL, F.S.A. London: 1848.
Harleian Manuscripts.
Cottonian Manuscripts. Titus, b. xi.
Lord BACON's History of Henry VII. Op. b. iii. London: 1740.
ANTHONY à WOOD. History and Antiquities of University, Oxon. 1674.
Publications of the Parker Society. 1846-53.

Professor Hecker's researches on the subject of the ravages of the disease in England, might not be uninteresting to our readers; who will then be in a position to follow us on some future occasion in a discussion of the nature of a malady, which five times within a hundred years devastated our island, and once, and once only, spread its ravages amongst the Teutonic races on the continent of Europe.

We may preface our historical *resumé* by noticing that the disease, in the form in which it then presented itself, was unknown before the year 1485, and that it has never reappeared since its last outbreak, in 1551. Its novelty gave it one of its appellations; it was called by the common people the "new acquaintance"; whilst its limitation to British soil gained for it on the continent the names of the King of England's Sickness, the English Sweating Sickness, *Sudor Britannicus.*

Characterised by the suddenness of its seizure, by its short and defined course of twenty-four hours, by its great fatality, by the profuse and fetid perspiration in which the patient was bathed, and from which the disease derived its most common name, by the frequency with which it attacked the same individual several times within a short period, or perhaps, we should more correctly say, by its relapsing tendency, by its selection of strong and robust men in the prime of life as its victims, by the equality with which it invaded the palaces of the rich and the cottages of the poor, we cannot wonder at its producing a marked effect on the national mind, and being long held in remembrance. Even as late as the days of the great rebellion, occasional references may be found to it in popular sermons and treatises; whereas we might have supposed its memory would have been effaced by the frequent outbreaks of plague which had intervened. The sweating sickness has come down to us as the remarkable epidemic of a remarkable age. In an era distinguished by the emancipation of thought, by the spread of letters, by the splendours of a social and religious reformation, death appeared in a new garb, and in unwonted tones asserted his dominion.

It has been a frequent observation, that epidemic diseases have had their origin in camps; and it is perfectly needless here to remind the reader of instances. Such will present themselves to every student of history. The English sweat is stated by Caius to have first appeared in the army of the Earl of Richmond, shortly after their landing; and doubtless they were predisposed by the circumstances of the expedition, by their confinement during their voyage in close, dirty ships, and especially by their previous habits (for they are described by

Philip de Comines as recruited from the loosest and most profligate class in Normandy), to suffer from any disease. But it is perfectly clear that, granting the distemper to have first appeared in the invading force, it was not long limited to it. It must quickly have spread amongst the population; as we learn from the *Historia Croylandensis* that, a few days after the landing of the earl, Lord Stanley excused himself from joining Richard III, by alleging that he was attacked by the new disease, he being then at his seat in Lancashire. A mere excuse, no doubt; but such as would not have been urged had not the progress of the epidemic rendered it possibly true. We likewise have proof that a fatal disease reigned at the time in York, although we lack information as to its precise nature. On the 16th of August, 1485, it was determined in the town council to send a messenger to King Richard with the offer of a force "for subduing of his enemies lately arrived in the partes of Wales". "Also it was determyned that all such aldermen and other of the counsail as was sojournyng, for the plage that reigneth, without the citie, should be sent for to give their best advises in such things as concerned the wele and savegard of the said citie, and all other inhabitants of the same" (Drake's *Eboracum*, b. i, p. 120). It is moreover remarkable, that, although the circumstances of the march of Richmond's army, and of its final struggle and victory, have come down to us with tolerable minuteness, no mention, as far as we are aware, is made by the chroniclers of any pestilence tracking their course. The battle was fought on the 22nd of August; and before the end of that month the epidemic appeared at Oxford, a town through which the army is not reported to have passed, and which, devoted to learning, may be supposed to have suffered less from military occupation than other places.

Whilst, however, the assertion that the malady commenced amongst the soldiery of the Earl of Richmond rests principally on the authority of Dr. Caius, who wrote his account three-quarters of a century after the event, yet, in the lack of other evidence, we believe we must receive it. Caius was evidently aware of the interest and importance of his subject, and would scarcely have hazarded such a statement had he not been assured of its truth. On the other hand, it is a groundless assumption to claim for the sweating sickness a foreign origin. No such disease had appeared in Normandy, Brittany, or elsewhere on the continent; and there is no reason for supposing other causes present to produce the first epidemic of 1485, than those which resulted in the outbreak of 1551, when it commenced at Shrewsbury, and importation from abroad was simply out of the question.

It was on the evening of the 1st of August, 1485, that the sails of Henry's little fleet were furled in the harbour of Milford Haven. They had accomplished the passage from Harfleur in seven days. The soldiers landed with promptitude, in the neighbourhood of the village of Dale, on the western side of the bay, and there encamped for the night. At sunrise the next morning they removed to Haverfordwest, a march of something less than ten miles. Here, reinforced by the men of Pembrokeshire, they proceeded to Cardigan, where they were joined by forces under Richard Griffith and John Morgan. Crossing the Severn, they entered Shrewsbury, where they were again augmented by a goodly band of Welshmen under Rice ap Thomas. The night before they entered the town, the army was encamped on Forton or Fortune Heath (to the west of Shrewsbury, near the river). They then marched to Newport, and the earl pitched his camp on a little hill adjoining, where he stopped a night. Here he was joined by the power of the young Earl of Shrewsbury, under George Talbot. He next halted at the town of Stafford, and thence marched on Lichfield, where his army bivouacked outside the walls. From this place they removed to Tamworth, their last halting-place before the great battle which decided the fate of England, and placed her crown on Henry's brow.

Three weeks were occupied in the march, and their road lay chiefly through a mountainous country, not, as far as we are aware, more likely to give origin to malarious influence than other parts of the island. Yet the halt of the army at Shrewsbury, the place at which the last outbreak of the "gret dethe and hasty" undoubtedly commenced; the passage of the river Severn, which in the year 1483, overflowing its banks, had inundated the whole of the surrounding country; and the encampment on the low marshy ground outside the walls of the city of Lichfield, are especially worthy of remark.

Fortune and victory sat on Henry's helm. Disbanding his army, he advanced by easy stages to London, greeted as he went by the acclamations of the populace. All things seemed to promise a

> "harvest of perpetual peace,
> By this one bloody trial of sharp war", when "sodenly", to use the graphic words of an old chronicler, "a newe kynde of sicknes came through the whole region, which was so sore, so peynfull, and sharp, that the lyke was neuer harde of to any mannes remembraunce before that tyme:

For sodenly a dedly and burnyng sweate inuaded their bodyes and vexed their bloud with a most ardent heat, infested the stomack and the head greuously : by the tormentyng and vexacion of which sicknes, men were so sore handled and so painfully pangued that if they were layed in their bed, beyng not hable to suffre the importunate heat, they cast away the shetes and all the clothes liyng on the bed. If they were in their apparell and vestures, they would put of all their garmentes, euen to their shirtes. Other were so drye that they dranke the colde water to quenche their importune heate and insaciable thirst. Other that could or at the least woulde abyde the heate and styntche (for in dede the sweate had a great and a strong sauoure) caused clothes to be layed upon theim asmuch as they coulde beare, to dryue oute the sweate if it might be. All in maner assone as the sweate toke them, or within a short space after, yelded vp their ghost. So that of all them that sickened ther was not one emongest an hundreth that escaped."

Consternation and affright reigned everywhere. "Some", says Caius, were "immediatly killed in opening theire windowes, some in plaieng with children in their strete dores, some in one hour, many in two it destroyed, and at the longest, to them that merilye dined, it gaue a sorowful Supper. As it founde them so it toke them, some in sleape some in wake, some in mirthe some in care, some fasting and some ful, some busy and some idle, and in one house sometyme three sometyme fiue, sometyme seuen sometyme eyght, sometyme more sometyme all, of the whyche, if the haulfe in euerye Towne escaped, it was thoughte great fauour." Numbers were seen rushing from their houses in a state of nudity, hoping to cool their burning torments. The general joy which the victory of Bosworth had inspired was changed into despondence and evil augury. With grim humour, the people exclaimed that the new reign must needs be one of labour, since it began with a sickness of sweat.

It was about the end of the month of August that the disease appeared at Oxford. Here, according to Anthony-à-Wood, it raged with violence for the space of six weeks, killing most of the students, or banishing them from the university. It would seem that it did not reach London until some days later. Several chroniclers state that the 21st of September was the date of its outbreak; yet, as Hecker suggests, it is probable that cases may have occurred before that time, although its virulence was not until then manifested. However this may be, it continued in the city until towards the end of October, but had sufficiently subsided to permit the coronation of Henry on the 30th of that month. During the time that the epidemic was at its height, the mortality was prodigious. On the 11th of October, the mayor, Thomas Hill, died; he was succeeded by Sir William Stokker, knight, who before eight days was also carried off. It was also fatal to several of the aldermen. Grafton says six; Stow enumerates four. The higher classes could claim no immunity from the common enemy. Many of the aristocracy, both secular and clerical, fell its victims. It is noticeable that this was the case in each succeeding epidemic.

From London and the eastern part of the kingdom, it spread to the western and southern districts, and did not wholly disappear until December. In this time it had invaded almost the whole kingdom—every town and village, says Grafton—but without crossing the Scottish border, or being conveyed to the sister kingdom of Ireland.

From the Croyland Annals, we learn that it carried off the excellent Abbot Lambert Fossdyke, after eighteen hours sickness. This is stated to have taken place on the 14th of November, although the writer in another place alters the date to the 14th of October; and we think the latter more probable, as, whilst we do not deny that the disease lingered, as Wood says, in some places until December, we should be inclined to suppose that the fury of the epidemic had in November and December partially subsided, and deaths consequently become rare. To this circumstance we are inclined in some degree to attribute the efficacy ascribed to the Anglican mode of treatment. But on this point we hope to touch hereafter.

Facts are wanting to give a minute topographical or numerical account of its ravages. Baines says that it prevailed in Lancashire; but he furnishes no particulars. We can only infer from general testimony the universality and magnitude of the evil. Its disappearance may have been consummated by a violent storm of wind, which prevailed on the 1st of the following January. For twenty-one years from this date, we read no more in English annals of a return of the "fereful tyme of the sweate."*

The kingdom was only recovering from the tremendous invasion of plague, which in 1499 carried off, it is said, in London alone 30,000 persons, and cessation from civil contention and foreign warfare promised increase and prosperity to her population, when, in the summer of 1506, the old enemy again started into existence. This epidemic appears generally to have been of a milder type, and deaths were in most places

* In 1491 and 92, a sweating plague is said to have prevailed in Ireland; according to the Annals of the Four Masters, its attack was of twenty-four hours duration. Ware says, but we know not on what authority, that it was brought out of England. (See Census of Ireland for the year 1851.)

unfrequent. We know little as to its origin or spread. As in the close of the first epidemic, the lessened mortality was ascribed rather to the effects of treatment than to any temporary diminution in the virulence of the disorder. One record has come down to us, which is sufficient of itself to show that, under favouring circumstances, the "new acquaintance" of 1506 was capable of being developed in all its ancient severity. In the Annals of Chester (Harl. MSS. No. 2125), we are told that in 1506 there died in one day, of the sweating sickness, three score and eleven householders, of whom only four or five were women. Another account says, that in three days there died ninety-one householders, four only being widows. It matters not which is correct; either is sufficient to prove that no real change had occurred in the nature of the sweating sickness. It lingered until the autumn, and then disappeared. Lysons and Hemmingways make the outbreak at Chester to have occurred in 1507; but Pennant, more correctly, as it appears to us, follows the date of 1506, given in the Chester Annals.

Eleven years elapsed, the crafty Richmond slept the sleep of death in the "sumpteous and solempne chapell which he had caused to be buylded", and his son reigned in his stead. Unexpectedly, in July 1517, the pestilence again raised its head. We believe that this sweat was the most fatal in its results of any of the series. The dismal scenes of the first epidemic were repeated. It "killed some within three hours", say the chroniclers, "some within two hours, some merry at dinner and dead at supper." "In some one town half the people died, in some other town the third part, the sweat was so fervent and the infection so great."

We learn incidentally, from a letter written by the Cardinal du Bellay, who was ambassador from France to Henry VIII, and himself a sufferer in the next epidemic of 1528, that it was estimated that 10,000 persons died in ten or twelve days. The context warrants us in the supposition that reference is made here to the metropolis alone. Taking this as a mere approximation, we shall at once see, by comparing it with the ravages of other epidemics, how frightful the mortality whilst it lasted was. In 1854, the total number of deaths from cholera and diarrhœa in London, extending over a period of six months, with a population of 2,517,048, was 14,806. The epidemic was at its height during the first fourteen days of September, when 4,371 persons were carried off. The mortality in the epidemic of 1849 was somewhat greater, viz. 18,036, the period again extending over several months. Even in the great plague year, 1665, when 68,590 persons died in London, and the city was nearly abandoned, the mortality never rose higher than 7,165 in a week; this number being reached in the third week of September. The population of the metropolis in 1676, is estimated in Graunt's Bills of Mortality at 384,000; consequently, in the year 1517, it must have fallen far short of 300,000. Making every allowance for exaggeration, supposing only one-half the number stated to have died in the time specified, the mortality for that time, taking into account the amount of population, must have been as great as in the worst irruption of bubo plague, and so appalling that, in the present day, we can form but a faint idea of it.

Rich and poor were equally victims. Rank claimed for its possessor no exemption; poverty was no shield. The deserted palace no longer echoed the sounds of mirth; the low wail of the mourner interrupted the silence of the streets. Henry VIII, a prince who, like Leviathan in the deep, seemed to consider the earth as merely formed to take his pastime therein, leaving the city, retreated with a few followers from place to place before the advancing waves of pestilence. His Court had been the seat of its triumphs. His private secretary, the learned Italian, Ammonius of Lucca, died a few hours after he had boasted to Sir Thomas More that by abstinence and regimen he had shielded himself and family. The Lord Grey of Wilton, the Lord Clinton, and many other of his knights, gentlemen, and officers, were no more. Michaelmas and Christmas passed without their usual festivities. No gathering of people was permitted, for fear of infection. Oxford and Cambridge, crowded with eager students, amongst whom were already germinating seeds which produced the Reformation, were again attacked, and the former was again deserted. The sweat continued until the middle of December; and its horrors were heightened by the supervention towards the winter of plague. In Chester the mortality from the combined diseases was so great, that grass grew a foot high at the town cross. England, again, with one remarkable exception, was *alone* the land of the shadow of death. The pestilence passed over to the town of Calais, at that period belonging to the British Crown. But here it is said to have attacked principally the English inhabitants; and we know that it not only did not spread through France, but (from a reliable source) that it did not even reach to Graveling.

It must have been during one of these earlier irruptions of the sweating disease, that a Latin prayer was composed, of which a copy has been preserved. It is addressed "ad beatum Henricum," either Henry the Emperor, who with his wife Cunegunde, were saints of the Romish calendar, or Henry VI.

of that name is intended, who was claimed as uncle by Henry VII., and who, his piety having nearly procured him canonization, was highly revered by the people. In it occurs the petition so characteristic of the period :—

"Non sudore,
Vel dolore,
Moriamur subito."

The whole is to be found in the *Gent. Mag.* for 1786, p. 747.

1528. We have now arrived at the fourth irruption of the disease, and fortunately can present a more detailed account of the historical facts connected with it, than we have been enabled to do whilst glancing at the three former epidemics. In this we shall necessarily correct a slight error into which Professor Hecker, from the paucity of his materials, has fallen. Although the mortality was not equal in magnitude to that of 1517, yet the influence was widely felt, the disease was distinguished by the same characteristics, and the deaths were quite numerous enough to be placed in comparison with those occurring in ordinary epidemic visitations. From the circumstance that the disease was again particularly rife in the Court, we have found many references to it in letters published under the Royal Commission in the "State Papers," and in similar collections. We propose illustrating our account with such extracts from these as may serve to bring before the reader a more definite picture of the prevailing state of things.

Hecker, following Grafton, states that the disease first appeared towards the end of May, in the most populous part of the city of London. This was not the case. Before its influence was felt in the capital, which was not until the 14th of June, it had been rife in the north. For Sir William Parre, writing to Wolsey, on the 31st of May, informs him that the Duke of Richmond had on account of its prevalence removed to Ledeston, in Yorkshire, three miles from Pontefract. It was brought out of Sussex into London, as we learn incidentally from an unpublished letter in the Cottonian Collection. We may therefore conclude that it had widely spread in the country districts during the latter part of May and the first weeks of June. Oxford, as usual, suffered severely. The rapidity with which it flew from district to district, and from town to town, obtained for it in 1551 the quaint name of the "posting sweat." A most graphic picture of the commencement of the epidemic in the metropolis, is given by the Cardinal du Bellay. We are indebted to Mr. Halliwell for the publication of this most interesting document, which forms part of the treasures contained in the Imperial Library of Paris. From it we shall now give our readers some extracts. The Cardinal's letter is dated London, June 18, 1528; he writes:—

"One of the filles de chambre of Mademoiselle de Boulen was attacked on Tuesday by the sweating sickness. The king left in great haste, and went a dozen miles off: but it is denied that the lady Anne Boleyn was sent away as suspected, to her brother the Viscount, who is in Kent. This disease, which broke out here four days ago, is the easiest in the world to die of. You have a slight pain in the head, and at the heart; all at once you begin to sweat. There is no need for a physician; for if you uncover yourself the least in the world, or cover yourself a little too much, you are taken off without languishing, as those dreadful fevers make you do. But it is no great thing, for during the time specified, about two thousand only have been attacked by it in London. Yesterday, having gone to swear the truce, they might be seen, as thick as flies, hurrying out of the streets and the shops into the houses, to take the sweat the instant they were seized by the distemper. I found the ambassador of Milan leaving his quarters in great haste, because two or three had been attacked by it."

"But to return to London. I assure you that the priests there have a better time of it than the physicians, except that there is not enough of them to bury the dead. If the thing lasts, corn will be cheap. Twelve years ago, when the same thing happened, 10,000 persons died in ten or twelve days, it is said, but it was not so sharp as it is now beginning to be. M. the legate (Cardinal Wolsey), had come for the term; but he soon had his horses saddled again, and there will be neither assignation nor term. Everybody is terribly alarmed." This is confirmed by Stow. The term was adjourned to Michaelmas.

From this account we see that at its first onset in London it seemed probable that the epidemic would be as fatal as its predecessor. This expectation was not realized. The mortality seems to have been unequal at different times during the same visitation. It did not gradually increase, as in the plague, to its maximum, and then as gradually diminish, but probably was never more fatal than at its first onset.

Henry's first retreat was Waltham in Essex, from which however he was speedily driven, by the seizure of the treasurer, two of the court ushers, and two of his valets de chambre. He immediately retired to Hunsdon in Hertfordshire, where he arrived on the 21st of June. News here reached him that Anne Boleyn, who had already become the object of his passion, was attacked by the disease. The occasion of this illness produced one of that remarkable series of love-letters, which have since become so celebrated, and the originals of which are preserved at Rome. In it he deplores her illness, states he would gladly bear half of it to have her cured, and regrets that he cannot send her his first physician, who was absent, but that in default of him he sends the second, "and the only one left, praying God that he may soon make you well, and then I shall love him more than ever." Happy indeed would it have been for the ill-fated Anne had the dart penetrated more deeply. The scene enacted in the "doleful prison in the Tower", on the 19th of May, 1536, would not then have disgraced the history of the English monarchy, and the escutcheon of the Tudor would have been spared one of its deepest stains!

The next document of any importance, in which we find reference to our subject, is a letter written by Brian (afterwards Sir Brian Tuke) to Cardinal Wolsey; and, risking the charge of prolixity, we cannot refrain from extracting from it a passage or two, as it exhibited bluff King Hal in the novel character of a medical adviser. Tuke dates from Hunsdon, June 23rd, 1528; and, in relating to Wolsey the particulars of a private interview he had with the King, respecting a letter he had received from the Cardinal, he thus writes:—"I red forthe til it camme to the latter ende, mencionyng Your Graces good comfort and counsail geven to His Highnes, for avoiding this infeccion, for the whiche the same, with a most cordial maner, thanked Your Grace: and shewing me, firste, a great proces of the maner of that infeccion; howe folkes wer taken; howe litel dangeir was in it, if good ordre be observed; howe fewe wer ded of it; howe Mastres Anne, and my Lorde of Rocheforde, bothe have had it; what jeopardie they have ben in, by retournyng in of the swet bifore the tyme; of the endevour of Mr. Buttes who hathe ben with them, and is retourned; with many other thinges touching those matiers, and finally of their perfite recovery; His Highnes willed me to write unto Your Grace, most hertily desiring the same, above al other thinges, to kepe Your Grace oute of al ayre, where any of that infeccion is, and that if, in on place any on fal sike thereof, that Your Grace incontinently do remove to a clene place; and so, in like cace, from that place to an other, and with a small and clene company: saying, that this is the thing, whereby His Highnes hathe pourged his house, having the same nowe, thanked be God, clene. And over that, His Highnes desireth Your Grace to use smal sowpers, and to drink litel wyne, namely that is big, and ons in the weke to use the pilles of Rasis; and if it comme in any wise, to swete moderately the ful tyme, without suffering it to renne in; whiche by Your Graces phisicians, with a possetale, having certein herbes clarified in it, shal facilly, if nede be, be provoked and contynued; with more good holsom counsail by His Highnes in most tender and loving maner geven to Your Grace, then my symple wit can suffise to reherse; whiche his gracious commaundement, I said, I wolde accomplish accordingly."

In the after part of his letter he informs the Cardinal that news had just arrived that Mr. Cary, whom he had shortly before met on his way to hunt, was "ded of the swet." "Our Lorde have mercy on his soule, and holde his hande over us." Proposing to join Wolsey, he tells him he dare not come through London, "wherfore I wol cost to the water side, and comme the rest by water, thorough London Bridge; though I promyse Your Grace there is non erthely riches shoulde cause me to travaile muche nowe, considering that the phisicians tel me ther is nothing, that more stirreth the mater and cause of the swet then moche traveil, and likewise commyng in the son."

In the city the ancient solemnity of the procession of the watch, on Midsummer eve, was discontinued, for fear of adding fuel to the spreading flame by collecting the populace: whilst the King removed to Hertford, at which place he was "moche troubled," for on the night of the 26th, there fell sick the Marquess and Marchioness of Dorset, Sir Thomas Cheney, Mistress Croke, Master Norris, and Master Wallop; who all, however, recovered; and Sir Francis Poyntz, who, says the writer of the letter we are quoting, "is departed, whiche Jhesu pardon." On these occurrences taking place, the King fled to Bishop's Hatfield in Hertfordshire.

On the 29th, we find Wolsey removing to Hampton Court, on account of the "vehement infection and sykenes, that ys fallen amonges his Graces folkes." Du Bellay's next letter, we shall see, gives a rather ludicrous account of the precipitate retreat of the "great child of honour," who, as all know from Cavendish, was dreadfully afraid of contagion, and used to carry with him an orange, stuffed with sponge steeped in vinegar and confections, against pestilent airs, the which he commonly held to his nose when he came to the presses, or when he was pestered with many suitors.

On the 30th the King had reached Tittenhanger in Hertfordshire, and here received news of the death of Sir William Compton, who was reported to be "lost by neclygens, in lettyng hym slepe in the begynnyng of his swete." No more on that day had been attacked in the Court, and those who had sickened on the 28th were recovered.

Grafton tells us that during the stay at Tittenhanger the place was daily purged with fires and other preservatives. An odd remedy against a sweating sickness at Midsummer!

The second letter of the Cardinal du Bellay is of this date; after recounting the names of nine courtiers who had been attacked, and of three who were dead, he says—"but when all is said, those who do not expose themselves to the air rarely die; so that out of more than 45,000 who have been attacked in London, not 2000 have died, whatever people may say. It is true that if you merely put your hand out of bed during the twenty-four hours, you instantly become stiff as a peacock. P.S. Since writing my letters, I have been informed that a brother of the Earl of Derby's, and a son-in-law of the Duke of Norfolk's, have died suddenly at the legate's (Wolsey), who slipped out at the back door with a few servants, and would not let any body know whither he was going, that he might not be followed. The king at last stopped about twenty miles hence, at a house which M. the legate has had built, and I have it from good authority, that he has made his will and taken the sacrament, for fear of sudden seizure. Nothing ails him, thank God!"

We shall hereafter see that the ambassador was inclined to think more seriously of it, when he had himself been a sufferer, and was the only survivor of nineteen who were attacked. However, in the absence of other evidence, we are bound to receive his statement as correct, in reference to the amount of mortality. And even this must have been proportionally as great as in our last epidemic of cholera and diarrhœa. It is calculated, in the Report of the Scientific Committee, published by the Board of Health, that seventy-one deaths in each 10,000 of the population of London took place, and that in this number there were 3473 cases of all forms of cholera and diarrhœa; in other words, that there was one death to every forty-eight attacks. But Du Bellay's statement gives an average of two deaths in forty-five seizures; or more than double the proportion. Again, it must be remembered that these occurred in the space of sixteen days, whereas the cholera epidemic lasted six months. We do not wish to be supposed to insist on this calculation; in either case it can merely be an approximation, and we advance it here only to show that even a mild epidemic of the sweating sickness was no slight pestilence.

On the first of July, we are informed that two cases had occurred at Tittenhanger; one being that of a gentleman's servant, the other, one of the King's wardrobe. On this day the King sends to Wolsey for "the byll that Mr. Fynche made, for the remedy of all suche as have fallyn syke in youre howse; for as His Hynes ys enformyd, he haythe doyne very well, boythe to bryng them to there swheyte ageine, when they fall owte, and allso to swayge the grete hete and burnyng."

On the 5th, we find the King again despatching to Wolsey to delay visiting him "untill the tyme be more propiciouse." In a former letter we learn that flying tales had reached Tittenhanger, that many of his Grace's folks were sick, and divers departed. Henry was as much frightened as the Cardinal, although on St. Thomas's day he sends him a message, in a letter written by Dr. Bell, to put away fear and fantasies, to commit all to God, and expresses a wish that "Your Grace's harte weer as gode as hys is." Both king and minister made their wills, and each took care that the assurance was conveyed to the other that he was not forgotten in the testament. In the letter of the 5th, the Cardinal is desired to "cawse generall processions to be made, unyversally thorough the realme, aswell for the good wetheringes, to thencrease of corne and fruyte, as also for the plage that now reignethe."

On the 9th, we find Henry preparing to remove from Tittenhanger to Ampthill in Bedfordshire, in consequence of the seizure of the "Lady Marques of Exeter," and commanding that all such as were with the Marquis and Marchioness should "departe in severall parcells, and so not contynue together."

On the 10th, the king had postponed his departure until the 11th; but, in the meantime, eight or nine had fallen sick, although none had been in jeopardy. The unpublished letter in the Cottonian Collection, before alluded to, bears date July the 14th. It is from Brian Tuke to Sir Peter Vannes. The disease had broken out in Tuke's house; and he says, "I write this at my waking after mydnyȝt, fearing to lye stil for the swet, with an aking and troubled hed." His wife had passed the paroxysm, but "veray weke," "and also sore broken oute about her mowthe and other places." His letter is principally filled with his opinion as to the causes and mode of spread of the epidemic. He allows that there is an infection, but believes that the disease is chiefly "provoked of disposicion of the tyme." He thinks that many frighten themselves into it. (How commonly we heard this in the late epidemics!) He flatters himself that he has obtained protection by the nightly use of a certain means; which, however he does not specify. The context would lead us to suppose that it was the application of cold in some form; for he says—"It wer to long a worke to declare unto you by what and howe I nyghtly put away the swet from me, and by what reason I dare do the same, when al other men take that so doing they kil them self." The chief facts of interest we learn from this letter are, that the sweat did not spread from Calais to Graveling, although there was constant intercourse between the two places, and that it was brought from Sussex into London. We may form some idea of his pathology by the following: "It is not so moch to be doubted to put away the swet in the begynnyng, and bifore a man's grese be well hote, keping molten, as it is taken. For though surely after the grese so heted it is no lesse but rather more danger for a man to take colde then it wer for an horse that in like case is destroyed."

On the 18th, the Abbess of Wilton in Wiltshire writes to Wolsey, that "it pleasithe Almyghty God to visite nowe the monastery with this greate plage of swetyng."

The French ambassador's third letter bears date the 21st, and from it we shall make our last extracts in reference to this visitation. He says: "As to the danger which is in this country, it begins to diminish hereabouts, but increases in parts where it had not been. In Kent it is rife at this moment." * * "The day that I had it at M. de Canterbury's (the archbishop), eighteen died of it in four hours; scarcely any escaped that day but myself, and I am not yet stout. The king has removed further than he was, and hopes that he shall not have the complaint. Still he keeps upon his guard, confesses every day, receives the sacrament on all holidays; and likewise the queen, who is with him. M. the legate does the same. The notaries have a fine time of it here: I believe there have been made a hundred thousand wills off hand, because those who died all went mad the instant the disorder became severe. The astrologers say this will turn to the Plague, but I think they rave."

The epidemic spread throughout the country, and in consequence of it the circuits of assize were adjourned. Ireland also now unquestionably felt its influence. In Cork it was very fatal; and in Dublin, in the month of August, the archbishop and many of the citizens fell victims. It continued in some parts of England until the autumn; for Magnus, writing to Wolsey from Sheriff Hutton in Yorkshire, on the 7th of October, says that in consequence of the "pestiferous and ragious swete," the Duke of Richmond has remained until now in a private place, with few attendants.

We must apologize to our readers for these lengthy details; but it is in descending to particulars we frequently can obtain that vivid impression of bygone events, which a mere general statement so often fails to convey.

In the following year, 1529, the sweating fever appeared at Hamburg, and spread throughout Germany, the Netherlands, Denmark, Sweden, and Norway. It is not within the limits of a historical paper, which is confined to an account of the sweating sickness in England, to trace the march of the pestilence. This has been most ably performed by Hecker. Wherever it appeared it was accompanied, as usual, by dread, death, and desolation. We cannot, however, agree with the German professor in his view of the production of the epidemic. We are inclined to avow ourselves contagionists, in a modified sense of the word. It is beyond doubt that the disease did not appear at Hamburg until, on the 25th of July, a ship arrived *from England*, commanded by a Captain Hermann Evers, on board which, several cases of sweating sickness had occurred. On the night of their landing four persons were attacked and died. It is true that the conflagration no longer spread widely in England, but it had not died out in the earlier part of the preceding winter, and we cannot but believe that its flickering embers still existed. Sporadic cases doubtless occurred, and even isolated outbreaks of the disease. At least, we have strong proof of one such taking place at Chester, in 1550, a year before the last great epidemic.* Hecker seems to think that the passengers on board Captain Evers's ship, acquired the sweat in the fogs of the German Ocean. But other ships must have been exposed to the same influence, and this was an isolated case. When, we would inquire, did a similar epidemic commence among the colliers of the Tyne, or the fishers of the Forth? The argument that he adduces from the fact that no sooner did report of the disease reach a place, than cases immediately occurred; and that, therefore, it spread more rapidly than by contagion, is the same advanced by our old friend, Brian Tuke, who says—"For when an hole man hath comen from London, and shewed of the swet, the same nyȝt al the toun, where the knowlege was, fal of it, and thus it spredeth yet as the fame roneth." What better proof of the intervention of human intercourse can we have than is given in this sentence? The solution of the problem lies in the "whole man who came from London." Evidently the rumour and the reality flew along the same conducting wire.

---

* A remarkable notice of the occurrence of the sweat in the town of Galway, in the year 1543, is given by Mr. Hardiman in his local history. The fact was obtained from "Town Annals", no longer accessible. We can only class it, as an isolated outbreak, with that of Chester in 1550. (Census of Ireland, 1851.)

Yet we would not insist too much on what after all must be matter of opinion. When we find the medical world of our own day so divided on the subjects of the spread of cholera and yellow fever, the facts of which appeal to their immediate observation, how can we hope to draw conclusions with certainty from the scant records of 300 years ago—at the best, but a faint glimmer to direct us through the darkness which surrounds the past? That an outbreak of the sweat occurred at Chester in the year 1550, is affirmed by all the local historians. The year seems fixed by the fact that the mayor, Edmund Gee, died of it. We have examined several lists of the mayors and sheriffs, both manuscript and printed; and they each place his mayoralty and death in the year 1550. The Chester Chronicles in the Harleian Collection, state that in the morning he left the pentice (a local court) in good health, and that he died before night. Forty persons are said to have been carried off in twenty-four hours. Of course we cannot positively declare that there has been no confusion of dates here; we only lay before our readers the unanimous testimony of the Chester authorities.*

We have now arrived at the fifth and last act of the tragedy. The final irruption of the sweating sickness commenced at Shrewsbury, in the year 1551, the fifth of the short but eventful reign of Edward VI. Caius and Stow name the 15th of April as the day of its first appearance, but a manuscript chronicle of the town dates its commencement from the 22nd of March. Local tradition yet points to the White Horse Shut, Frankwell, as the focus from which the malady spread. Hecker, without sufficient ground, places the amount of mortality at 960. But Caius, whom he follows, merely states that in one city (*unâ civitate*) that number died. We are inclined to doubt, with the authors of the History of Shrewsbury, whether, as has been generally affirmed, Caius was present in that town at all. When he says, "Ipse dum hæc tragedia agebatur, præsens spectator interfui," he only states that he was an eye-witness of the dreary spectacle; and

* The manuscript above quoted, making the last Chester outbreak to have occurred in 1550, places the first in 1506. If we believe these annals to be incorrectly dated by a year, in that case the true date of the earlier visitation will be 1507, as given by several writers. The affirmation of Caius, that the disease appeared at Westchester (the old name for Chester) in 1551, favours this assumption. On the other hand, the Vale Royal, Ormerod, Hemingways and Lysons all agree in stating that 1550 was the year in which the town was severely visited by the malady. Whichever view we take, it would appear that one of the Chester visitations must have occurred in a year (1507 or 1550) not marked by a general epidemic, unless we gratuitously fix a charge of incorrectness on the early local annalists.

there are reasons which render it more probable that he observed it in London than at Shrewsbury. However this may be, we have his testimony that it spread from its place of origin to Ludlow, Presteign, and other places in Wales, thence to Westchester, Coventry, Drenfoorde (?), and the south, before it came to London, which it reached on the 7th of July, three months after its first appearance. He gives a most vivid description of the consternation, horror, and desolation that reigned. Business was at an end; citizens fled to the country; peasants thronged the towns; many sought an asylum in foreign lands. The shrieks of women, rushing half naked from their habitations, mingled with the groans of the dying, and the deep clang of the funeral bell, booming through the misty air from every tower and steeple, deafened the ear, and struck terror to the heart of the passer. The epidemic was at its height in the capital from the 9th to the 19th of July, and it lingered until the 30th. In this time, at the lowest computation, nearly a thousand people perished. The exact number is somewhat differently stated, Stow says 960 died, of whom 800 in the first week. Caius (English treatise) reports that 761 died from the 9th to the 16th, besides those on the 7th and 8th, of whom no register was kept, and 142 from the 16th to the 30th. Machyn, a citizen resident in London, says that 872 were certified by the chancellor to have perished from the 8th to the 19th; whilst, in a manuscript in the Harleian Collection, we are told that 938 persons were carried off between the 7th and 20th. These numbers render it probable that when Caius, in his Latin treatise, written some time after, speaks of 960 dying in one city, his statement refers to the metropolis. One testimony, however, places the mortality much higher. Christopher Froschover, in a letter, dated London, August the 12th, affirms that 2,000 had died in the city, and 200 at Cambridge. The short space of time occupied by the pestilence, with the awfully abrupt seizure, and speedy termination of the fatal cases, rendered the destruction so appalling. It was the "sudden death," with battle and murder equally dreaded.

Again the palace was attacked. A celebrated lawyer, Sir Thomas Speke, was seized there, and had only time to reach his house in Chancery Lane, before he breathed his last. There were some dancing in the Court at nine o'clock, who were dead at eleven, says a sermon of the period. There died in London, writes Machyn, "mony marchants and grett ryche men and women, and yonge men." Howes, in his continuation of Stow, relates that "seven honest householders did sup together, and before eight of the clock the next morning six of them were dead." The young king fled to Hampton Court, whence he addressed a letter to the bishops, inciting them to persuade the people to prayer, and to see God better served. There are several references to the malady in the preaching of Bradford and Hooper: the latter made it the subject of a pastoral charge and homily.

We are unable to trace the pestilence from town to town, but sufficient data have been collected to shew how widely the destructive principle was disseminated. In June we find it at Loughborough, in Leicestershire. In the parish register is the curious entry: "1551, June. The swat, called New acquaintance, alias Stoupe knave and know thy master, began on the 24th of this month." It was in July that the disease appeared at Cambridge. Pursuing their studies in the University, were the young Duke of Suffolk, and his brother, Charles Brandon, equally distinguished for ability, worth, and learning. Alarmed by the outbreak, they hastened, with a few attendants, to Kingston, five miles distant. Here their chosen friend and companion, Charles Stanley, was seized, and expired in ten hours. In sorrow and consternation the brothers fled to the Bishop of Lincoln's palace at Bugden, in Huntingdon, where they were joined, late at night, by their mother. Scarcely had she embraced them, when the Duke was attacked by the fatal symptoms, and in five hours, despite the endeavours of physicans, ceased to breathe. Within half an hour the younger brother, who slept in a distant part of the palace, was also a corpse. Their deaths created universal sorrow, the more, perhaps, that, through the influence of their mother, they were known to be attached to the principles of the Reformation. Our account is extracted from the very rare and interesting black-letter tract by Sir Thomas Wilson.

Late in July the pestilence was at Gloucester, whence Bishop Hooper, in a letter, dated August 1st, writes: "After I had begun this letter, my wife, and five others of my chaplains and domestics, were attacked by a new kind of sweating sickness, and were in great danger for twenty-four hours. I myself have but recently recovered from the same. The infection of this disease is in England most severe." At Bristol the mortality was great. It lasted from Easter to Michaelmas, and several hundreds are said to have been carried off every week. Small towns and villages equally felt the influence. The parish register of Uffculme, in Devonshire, records that of thirty-eight deaths occurring in 1551, twenty-seven were in the first eleven days of August, and sixteen of them in three days. These persons are said to have died of the "hote sickness or stup gallant." (This latter name is evidently derived from the *Trousse Galant* of the French, a disease which had been epidemic in France in 1528, and afterwards, and which, we would suggest, was allied to the worst form of scarlatina.)*

Whilst the south thus suffered, the north could offer no asylum. In York and Hull the pestilence was severely felt. It ravaged Lancashire; one parish register gives us the dates and number of deaths. In Ulverstone parish there were five buried on the 17th, two on the 18th, four on the 19th, eleven on the 20th, six on the 21st, six on the 22nd, two on the 23rd, and three on the 24th of August. On the 7th of that month we find it in the neighbourhood of Leeds. Whitaker quotes that "on the 7th of August, 1551, the sweating sickness was so vehement in Liversage, that Sir John Neville was departed from Liversage Hall to his house at Hunslet, for fear thereof. It speedily despatched such as were infected; for one William Rayner, the same day he died, had been abroad with his hawk."

The disease did not disappear till the end of September. Several of the most distinguished men of the age fell its victims, as we learn in a letter from Roger Ascham to Sir William Cecil. In Catholic countries the sad fate of England was held a judgment on her departure from the Romish faith. At home it roused that spirit of piety and benevolence, which is never wanting in the Anglo-Saxon race in the time of suffering and distress. The religious fervour of the period burned higher in the gale; and, no doubt, amid the terrors of the sweating sickness, many acquired that trust in Providence and fearlessness of death, which were in the ensuing religious troubles to be so severely tried. On the other hand, amongst the masses, as Grafton drily observes, "As the disease ceased, so the devotion quickly decayed."

From this time the Sudor Britannicus has never reappeared in its epidemic form. In one or two instances, we have seen isolated notices of death occurring from sweating sickness. But we have no means of judging the nature of the disease referred to under that name, or of determining the credibility of the statement. One thing is certain: no large district of our island has ever been ravaged by its indigenous pestilence, since the memorable year in which the destroying angel alighted on the sedgy banks of the gentle Severn.

*Francis C. Webb.*

---

* It was a fatal inflammatory fever, followed in the survivors by loss of hair and nails, and dropsical effusions.

